# Supplementary material for: Subgroup fairness in two-sided markets
Source: PLoS One. 2023 Feb 22;18(2):e0281443. doi: 10.1371/journal.pone.0281443 (PMC9946267; doi:10.1371/journal.pone.0281443)
Supplement: S1 Appendix — (PDF) [file pone.0281443.s001.pdf]

S1 Appendix

Cross validation

Since batch contains 10 jobs and 20 workers, there might be concerns about whether the batch of jobs can capture the behaviour of the whole dataset of NYC Taxi. We call it a trial if the same batch is used in all implementations. For each trial, a new batch is randomly chosen from the whole dataset.

We repeat the same experiments in Fig 2 for k-fold cross validation, which is usually performed with k = 5 or k = 10 [1, 2]. Firstly, we consider $k = 5$, such that we need to implement five fold. Fig 2 displays the result of Fold 1. S1 Fig presents the trade-off plots of the four other folds (i.e., Fold 2-5). Each dot represents one implementation of augmented-Lagrangian formulation (10) in `tssos`. Red dots denote the experiment of "Sühr et al. 2019 (L)". Our method Intra 5 + Inter 3 (L) is denoted by green dots. The position of each dot represents the value of Intra-fairness and Inter-fairness from the experiment results. The Pareto front is shown by a black curve. The procedure is in Algorithm 1. Further, we implement another 10-fold cross validation (i.e., $k = 10$) following the same algorithm. The results of 10 new folds are presented in S3 Fig.

Furthermore, we implemented the formulation Intra 5 + Inter 3 (L) ($\gamma^{(1)} = \gamma^{(2)} = 0.5$) for 100 trials with one run in each trial. In S2 Fig, we give the distributions of Intra-fairness and Inter-fairness trade-off from the 100 trials. Explicitly, each green dot represents the measures of Intra-fairness and Inter-fairness of one trial. The histograms on the top and on the left side show the distribution of measures of Intra-fairness and Inter-fairness across the 100 trials. Fig 3 is a subfigure in S2 Fig. See Algorithm 2.

Algorithm 1 Cross validation (5 or 10 folds)

Input: $N = 5$ or 10 (The number of folds); $|\mathcal{C}| = 10$; $|\mathcal{D}| = 20$; NYT Taxi Dataset.
Output: $N = 5$: Fold 1 in Fig 2 and the rest (Fold $2 - 5$) in S1 Fig.
$N = 10$: Fold $1 - 10$ in S3 Fig.

for Fold $n = 1, \ldots, N$ **do**
  Randomly pick a batch of jobs from the dataset with size of $|\mathcal{C}|$.
  Implement formulation "Intra 5 + Inter 3 (L)" in (10) with parameters $\gamma^{(1)} = 0.5, 0.6, \ldots, 0.9$, $\gamma^{(2)} = 1 - \gamma^{(1)}$ and $\gamma^{(3)} = 0$, for 5 trials. (5×5 runs in total)
  Implement formulation "Sühr et al. 2019 (L)" in (10) with parameters $\gamma^{(1)} = 1, \gamma^{(2)} = \gamma^{(3)} = 0$, for 25 trials. ($25 \times 1$ runs in total)
  From each run, calculate measures of inequality, i.e., Intra 2 ($\text{GE}(1)_t$), Intra 3 ($\text{GE}(0)_t$), Intra 4 (Gini_t), and Inter 1, Inter 2, Inter 3.
end for
Make a 3×3 trade-off plot of every pair of Intra- and Inter-fairness for each fold.

Algorithm 2 Cross validation (100 folds)

Input: $N = 100$ (The number of foldss); $|\mathcal{C}| = 10$; $|\mathcal{D}| = 20$; NYT Taxi Dataset.

Output: The trade-off plots in S2 Fig.

1: **for** Fold $n = 1, \ldots, N$ **do**

2: Randomly pick a batch of jobs from the dataset with size of $|\mathcal{C}|$.

3: Implement formulation "Intra 5 + Inter 3 (L)" in (10) with parameters $\gamma^{(1)} = \gamma^{(2)} = 0.5$ and $\gamma^{(3)} = 0$, for 1 trial.

4: From the results of this trial, calculate measures of inequality:

- Intra-fairness: Intra 2 ($\mathrm{GE}(1)_t$), Intra 3 ($\mathrm{GE}(0)_t$), Intra 4 (Gini_t).
- Inter-fairness: Inter 1, Inter 2, Inter 3.

5: **end for**

6: Make a single 3×3 trade-off plot of each pair of Intra- and Inter-fairness in S2 Fig.

References

1. Kuhn M, Johnson K, et al. Applied predictive modeling. vol. 26. Springer; 2013.
2. James G, Witten D, Hastie T, Tibshirani R. An introduction to statistical learning. vol. 112. Springer; 2013.
3. Sühr T, Biega AJ, Zehlike M, Gummadi KP, Chakraborty A. Two-sided fairness for repeated matchings in two-sided markets: A case study of a ride-hailing platform. In: Proceedings of the 25th ACM SIGKDD International Conference on Knowledge Discovery & Data Mining; 2019. p. 3082–3092.
