# Supplementary figures and images for: Subgroup fairness in two-sided markets
Source: PLoS One. 2023 Feb 22;18(2):e0281443. doi: 10.1371/journal.pone.0281443 (PMC9946267; doi:10.1371/journal.pone.0281443)

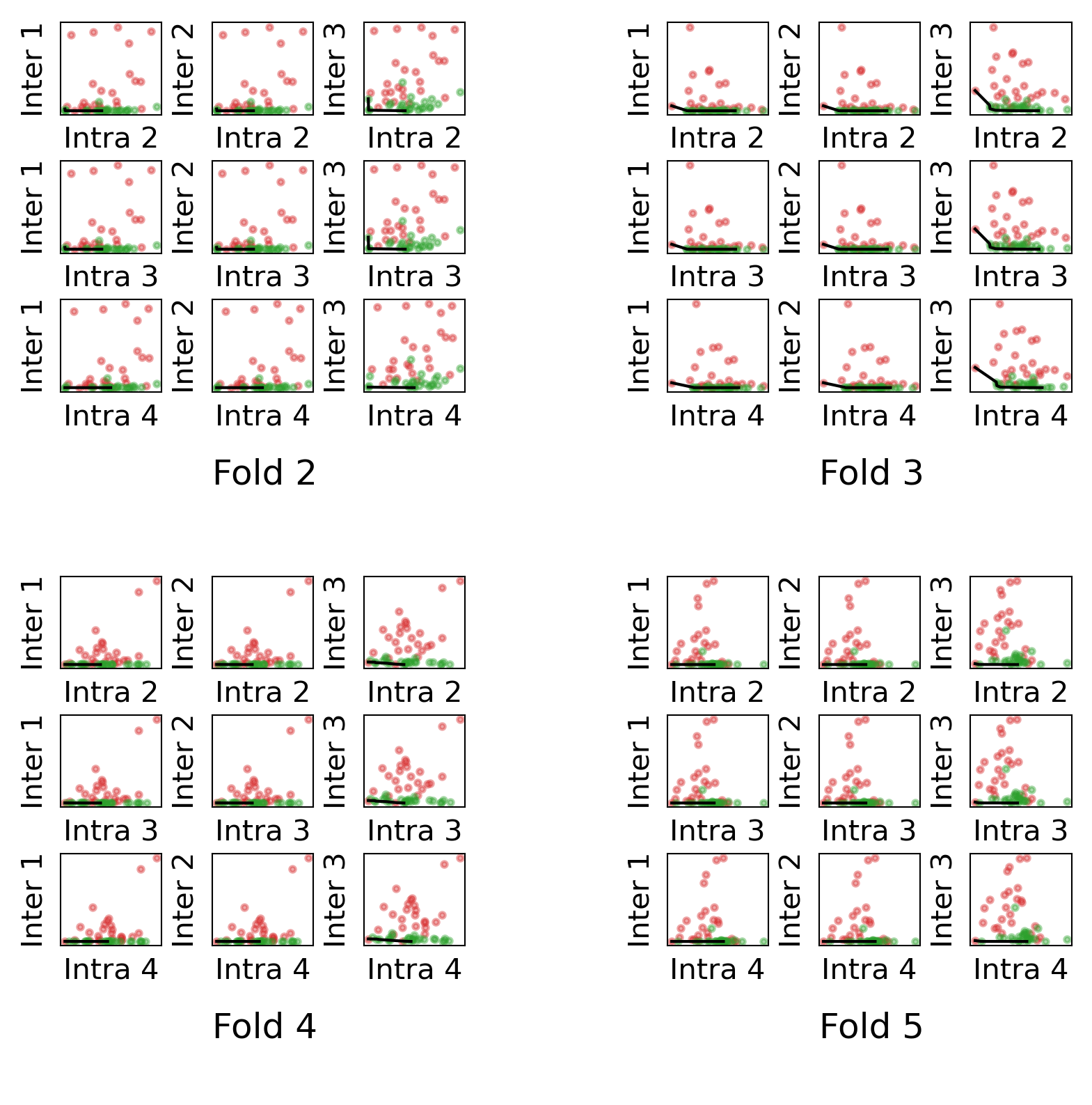

Supplement: S1 Fig — Fold 1 is presented in Fig 2. Each subplot presents trade-off between a pair of Intra- and Inter-fairness with Pareto-Front. Each green dot represents one trial of the implementation of augmented Lagrangian formulation (10) in tssos. Red dots denote a trial of “Sühr et al. 2019 (L)”. The experimental details are in S1 Appendix. (TIF) [file pone.0281443.s002.tif]

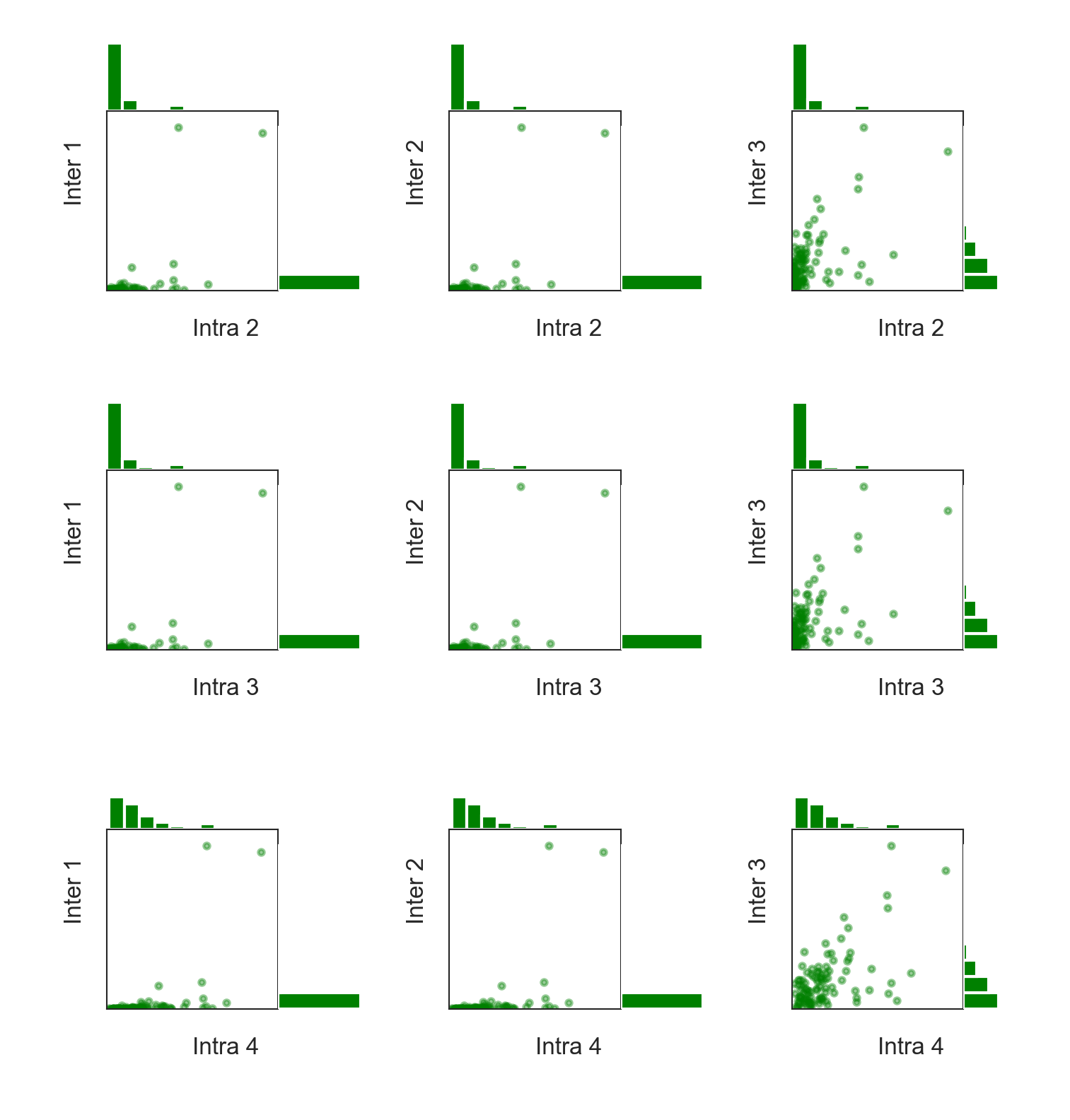

Supplement: S2 Fig — In each subplot, each dot represents the values of corresponding Intra-fairness and Inter-fairness notions of one trial. The histograms on top and on left side show the distribution of Intra-fairness and Inter-fairness of the 100 trials. The experimental details are in S1 Appendix. (TIF) [file pone.0281443.s003.tif]

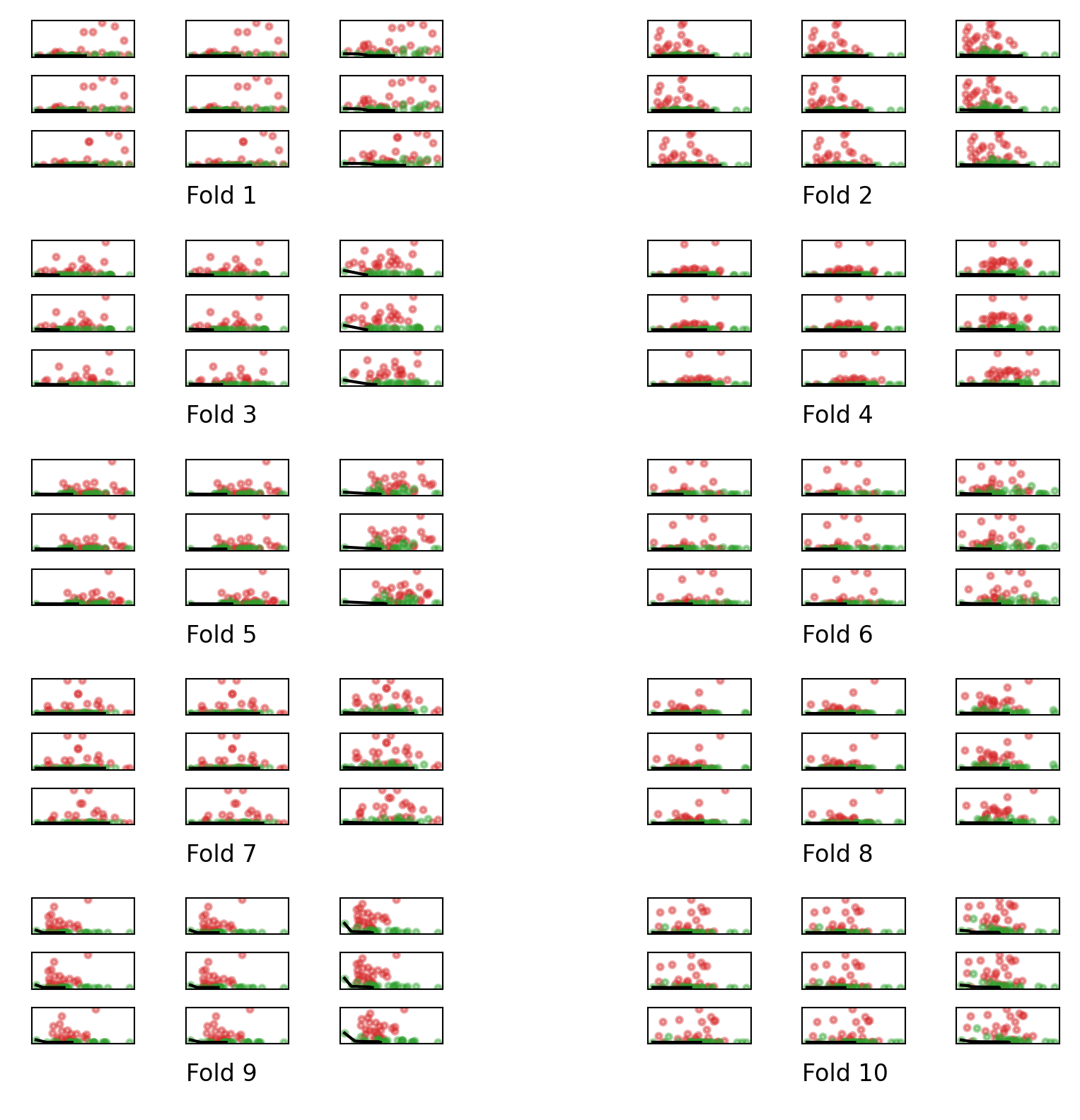

Supplement: S3 Fig — We show the trade-off plots of 10 new trials. (TIF) [file pone.0281443.s004.tif]
