# Supplementary material for: Subgroup fairness in two-sided markets
Source: PLoS One. 2023 Feb 22;18(2):e0281443. doi: 10.1371/journal.pone.0281443 (PMC9946267; doi:10.1371/journal.pone.0281443)
Supplement: Attachment — Submitted filename: response letter.pdf [file pone.0281443.s005.pdf]

Dr. Ashwani Kumar
Academic Editor, PLOS ONE

4. September 2022

Dear Dr. Kumar, Dear Reviewers, Dear Editorial Team,

Re: "Subgroup Fairness in Two-Sided Markets" [PONE-D-22-04438]

Please allow us to once again thank the Reviewers and the Editorial Team for the time taken to review our paper, and for the thoughtful feedback provided. We found the comments to be extremely useful, and have revised our paper accordingly.

We have now completed our revision, and we would be grateful if the enclosed, revised manuscript could be considered for publication in PLOS ONE. We have enclosed the new version of our manuscript with this letter. In the revised version of our manuscript, the revisions suggested by the Reviewers have been highlighted in blue font. We have also included our detailed responses to the Reviewers' comments in with this letter, below.

We thank the Reviewers once again for their comments. We believe that the paper is a significant improvement over the previous manuscript and we hope that the paper can now be accepted for publication.

Yours respectfully,

Quan Zhou (on behalf of the coauthors)

Journal Requirements:

When submitting your revision, we need you to address these additional requirements.
1. Please ensure that your manuscript meets PLOS ONE's style requirements, including those for file naming. The PLOS ONE style templates can be found at `https://journals.plos.org/plosone/s/file?id=wjVg/PLOSOne_formatting_sample_main_body.pdf` and `https://journals.plos.org/plosone/s/file?id=ba62/PLOSOne_formatting_sample_title_authors_affiliations.pdf`

We have re-formatted the manuscript to meet PLOS ONE's style requirements.

2. We note that the grant information you provided in the 'Funding Information' and 'Financial Disclosure' sections do not match. When you resubmit, please ensure that you provide the correct grant numbers for the awards you received for your study in the 'Funding Information' section.

Thank you for this comment. We have now corrected this issue.

3. Thank you for stating the following in the Acknowledgments Section of your manuscript: "Quan's and Bob's work has been supported by the Science Foundation Ireland under 379 Grant 16/IA/4610. Jakub acknowledges support of the OP RDE funded project 380 CZ.02.1.01/0.0/0.0/16 019/0000765 "Research Center for Informatics". We note that you have provided additional information within the Acknowledgements Section that is not currently declared in your Funding Statement. Please note that funding information should not appear in the Acknowledgments section or other areas of your manuscript. We will only publish funding information present in the Funding Statement section of the online submission form. Please remove any funding-related text from the manuscript and let us know how you would like to update your Funding Statement. Currently, your Funding Statement reads as follows:

"The funders had no role in study design, data collection and analysis, decision to publish, or preparation of the manuscript."

Please include your amended statements within your cover letter; we will change the online submission form on your behalf.

We have removed the acknowledgements section. Please add the funding statement as follows:

> *The work of Quan Zhou and Rober Shorten has been supported by the Science Foundation Ireland under 379 Grant 16/IA/4610. Jakub Mareček acknowledges support of the OP RDE funded project 380 CZ.02.1.01/0.0/0.0/16 019/0000765 "Research Center for Informatics".*

Response to Reviewer #1:
I have now completed the review of the manuscript titled "Subgroup Fairness in Two-Sided Markets". The manuscript have introduced the notion of Inter-fairness across subgroups in a two-sided market, in addition to Intra-subgroup fairness, among individuals within a subgroup. I have some suggestions to further improve the quality of the manuscript.

1. The introduction section requires some relevant articles which used ML and DL for various applications [1-6], and also include some more market fairness applications using ML. Please add these in the related work section.

We have added a paragraph of the applications of AL and ML in related work. We have added material related to fairness rather than the paper on environmental modelling. Please see blue texts in Section of Related work. We thank the reviewer for this comment.

2. Please provide the computational complexity of all the models, see CDLSTM, SMOTEDNN, DNNBOT, PCCNN etc.

While the models of CDLSTM, SMOTEDNN, DNNBOT, and PCCNN do not consider fairness, the best we could do is to explain that these are models without any fairness considerations, wherein can obtain critical points of unclear value using first-order methods. We imagine that it may be preferable to maintain silence and decorum.

4. What is the future scope of the proposed research, authors have described the limitations in good way, I suggest that these can be the future scope of the work.

We have added a paragraph suggesting ideas for future work. Please see blue texts in Section of Conclusion. We thank the reviewer.

5. These days ML and AI are utilized to solve other applications which are based on several parameters, I suggest to make a small paragraph which discusses the role of AI and ML methods, authors can use some of the references provided in the comments 1 and 2.

We have added a paragraph on applications of AL and ML in related work. This paragraph directs the reader to background work on applications of AI. Please see blue texts in Section of Related work.

6. Cross validation need to be clarified, why 5 is chosen, pl see [7].

5-fold cross validation is typical of that used in industry. Indeed, many books and standard references suggest the use of 5-fold validation. For example: (James et al. 2013, p. 184) have explained:

> *To summarize, there is a bias-variance trade-off associated with the choice of k in k-fold cross-validation. Typically, given these considerations, one performs k-fold cross-validation using $k = 5$ or $k = 10$, as these values have been shown empirically to yield test error rate estimates that suffer neither from excessively high bias nor from very high variance.*

As stated by (Kuhn and Johnson 2013, p. 70),

> *The choice of k is usually 5 or 10, but there is no formal rule. As k gets larger, the difference in size between the training set and the resampling subsets gets smaller. As this difference decreases, the bias of the technique becomes smaller.*

Accordingly, k-fold cross validation is usually performed with $k = 5$ or $k = 10$. We have chosen the former one. These references have been added to the manuscript.

Response to Reviewer #2:
This paper formulates the objective of the market-clearing problem as a weighted sum of measures of subgroup fairness (Inter-fairness), fairness within each subgroup (Intra-fairness), as well as utility for the customers (Customer-Care). The authors use a non-convex augmented Lagrangian relaxation of the objective and show that it can be approximated to any precision in polynomial time with respect to the number of market participants. Finally, the efficacy and scalability of the approach are demonstrated and different trade-offs between the objective's terms are explored on a taxi trip dataset.

Thank you!

Strong points:
* The variant of the market-clearing problem addressed in this paper is of considerable importance for understanding and promoting fairness in two-sided markers given the recent rise of the gig economy.
* Despite the non-convexity of the formulated objective (due to the inclusion of non-linear terms), the authors show that such an objective can be optimized by approximating an augmented Lagrangian relaxation that considers the violations squared of the objective's linear equality constraints. Moreover, the optimization can be carried out to any fixed precision in polynomial time. This allows for efficient computation of the proposed market-clearing mechanism.
* Comprehensive experimentation has been conducted on the well-known 2018 NYC Yellow Taxi Trip Dataset, the results of which suggest that (1) the proposed approach is effective and scalable, and that (2) a measure of inter-fairness can be included in the objective function without compromising intra-fairness.
* The formulation of the inter-fairness measure is general enough in the sense that it can easily be extended to cases with multiple subgroups or overlapping subgroups of sensitive attributes.
* The paper is well organized and written in a quite clear and comprehensive manner. Also, the used notation is clear and consistent throughout the entire paper.

Thank you!

Weak points:
* At the beginning of Section 5, the authors claim that they used "SDPA as an SDP solver via the tssos library" that "provides global optima of Figure 1 in the worst case in polynomial time". Nevertheless, I am slightly confused by this claim as how can one guarantee that the global optima would be found considering that the original objective and its augmented Lagrangian relaxation are non-convex?

The reviewer may recall the concept of "hidden convexity", i.e., that there are non-convex optimization problems that are solvable in P (polynomial time). Let us recall two textbook examples of hidden convexity.

- *First, consider the search for a shortest path in a digraph. Given a directed graph* (V, A) *with source node* s*, target node* t*, and cost* w_{ij} *for each edge* $(i, j) \in A$*, consider the integer linear program with binary variables* x_ij*:*

$$\min_{x_{ij}\in\{0,1\}} \sum_{i,j\in A} w_{ij}x_{ij} \ s.t. \ \sum_j x_{ij} - \sum_j x_{ji} = \begin{cases} 1, & \text{if } i = s; \\ -1, & \text{if } i = t; \\ 0, & \text{otherwise.} \end{cases} \quad (1)$$

  Due to the fact that the variables x_{ij} *are restricted to binary values, the feasible set is non-convex. There is, however, convexity hidden in the fact that every basic optimal solution of the linear programming relaxation* $0 \le x_{ij} \le 1$ *(when one exists) has all variables equal to 0 or 1, and the set of edges whose variables equal 1 form an s-t dipath. See L. Taccari ("Integer programming formulations for the elementary shortest path problem," European Journal of Operational Research 2016) for further details.*

- *Consider, as another example,* $\max x$ *s.t.* $x^2 + y^2 = 1$*. The feasible set is a circle, which is non-convex (unlike the disc, which is). At the same time, it is easy to see that the variable* y *is redundant. We can simplify the problem to* $\max x$ *s.t.* $0 \le x \le 1$ *and it becomes clear the solution is* $x = 1, y = 0$*.*

Returning to our setting:

- *The original non-convex problem (8) may or may not be NP-Hard. We make no claim on its complexity or the computability of global optima in P.*
- *However, its non-convex relaxation (10) is equivalent to a linear semidefinite program, as shown by S. Poljak, F. Rendl, and H. Wolkowicz ("A recipe for semidefinite relaxation for (0, 1)-quadratic programming," Journal of Global Optimization, vol. 7, no. 1, pp. 51–73, 1995). We can hence claim to provide, up to any precision, an approximation to the global optimum of the non-convex relaxation (10), in polynomial time, by solving the linear semidefinite program.*

We believe that this is rather a nice, non-trivial example of hidden convexity.

* The trade-off parameters $\gamma^{(1)}, \gamma^{(2)}$ and $\gamma^{(3)}$ are fixed throughout the experiments. I am wondering why the authors did not consider to automatically optimize the values of these parameters in addition to the other weights? Although the authors were able to essentially "grid-search" through various combinations of the trade-off parameters on the Yellow Taxi Trip dataset; using the same exploration strategy to determine the trade-offs on larger datasets would be quite challenging and computationally intensive. In their response, I would encourage the authors to elaborate a bit on the possibility of learning/optimizing the trade-off parameters automatically.

Thank you for your suggestion. This could easily have been incorporated as part of a sensitivity/robustness analysis. However, while important, we felt the core message of the paper is one of fairness, and the fairness metrics, and that a sensitivity analysis of the overall system design is a subject best discussed in detail in the context of a specific use-case. Consequently, we have included it in our ideas for future work. Please see blue texts in Section of Conclusion. We hope that this is acceptable.

* There are also a few minor points that require attention: - Page 11, line 292: Consider replacing “independent T test” with “independent t-test”.

We have replaced all “independent T test” with “independent t-test”.

- Figure 1 is not essentially a figure as such but rather a reformulation of the optimization problem. I believe that it should be marked and referenced as an Equation rather than a Figure. Thus, I would suggest replacing Figure 1 with an equation label.

We have marked Figure 1 as an equation.

- In the discussion of the experiments, Figure 4 is referenced and discussed before discussing Figure 3. In that case, the order of appearance of the two figures should be reversed.

We have moved Figure 4 forwards.

Overall, the paper is well developed and seems to be a good fit for PLOS One. Although there are some weak points that require certain revisions to be made, I believe that the strengths of this paper outweigh its weaknesses.

Thank you!
