# Supplementary material for: Subgroup fairness in two-sided markets
Source: PLoS One. 2023 Feb 22;18(2):e0281443. doi: 10.1371/journal.pone.0281443 (PMC9946267; doi:10.1371/journal.pone.0281443)
Supplement: Attachment — Submitted filename: Response to Reviewers.pdf [file pone.0281443.s006.pdf]

Dr. Ashwani Kumar
Academic Editor, PLOS ONE

22. November 2022

Dear Dr. Kumar, Dear Reviewers, Dear Editorial Team,

Re: "Subgroup Fairness in Two-Sided Markets" [PONE-D-22-04438]

Please allow us to once again thank the Reviewers and the Editorial Team for the time taken to review our paper, and for the thoughtful feedback provided. We found the comments to be extremely useful, and have revised our paper accordingly.

We have now completed our revision, and we would be grateful if the enclosed, revised manuscript could be considered for publication in PLOS ONE. We have enclosed the new version of our manuscript with this letter. In the revised version of our manuscript, the revisions suggested by the Reviewers have been highlighted in blue font. We have also included our detailed responses to the Reviewers' comments in with this letter, below.

We thank the Reviewers once again for their comments. We believe that the paper is a significant improvement over the previous manuscript and we hope that the paper can now be accepted for publication.

We have included the updated statement in cover letter. Our figure files have been corrected by PACE digital diagnostic tool.

Yours respectfully,

Quan Zhou (on behalf of the coauthors)

Response to Reviewer #1: Dear Authors I observed that the authors have their own perspective around fairness only. I am also surprised to see the response to the comments which again shows the narrow context drawn by authors to improve the clarity on validation, which is highly important for studies which used computation in any aspect or application.

We have extended our experiments in cross validation and robustness analysis in Section 5 and Supporting information.

The response such as [...] "the choice of k is usually 5 or 10, shown very limited perspective.

As we have explained earlier, 5-fold cross validation is typically used in industry. Indeed, many books and standard references suggest the use of 5-fold validation. For example: (James et al. 2013, p. 184) have explained:

> *To summarize, there is a bias-variance trade-off associated with the choice of k in k-fold cross-validation. Typically, given these considerations, one performs k-fold cross-validation using $k = 5$ or $k = 10$, as these values have been shown empirically to yield test error rate estimates that suffer neither from excessively high bias nor from very high variance.*

As stated by (Kuhn and Johnson 2013, p. 70),

> *The choice of k is usually 5 or 10, but there is no formal rule. As k gets larger, the difference in size between the training set and the resampling subsets gets smaller. As this difference decreases, the bias of the technique becomes smaller.*

These references have been added to the manuscript. On the other hand, we have conducted both $k = 5$ (Fig 2 & S1 Fig) and $k = 10$ (S3 Fig).

Response to Reviewer #2: The authors have addressed the weak points suggested in my previous review, provided a point-by-point response to each and revised the manuscript accordingly. Therefore, I would suggest that this revised version is considered for publication in PLOS One if the other reviewers share the same view.

Thank you!

Response to Reviewer #3: The research content of this manuscript is substantial. SSubgroup Fairness in Two-Sided Marketsïs a research topic with economic-value and social-value.

Thank you!

The manuscript provided many descriptions and analyses of the experimental result. However, this method or algorithm lacks robustness analysis and generalization analysis.

We have added a discussion of robustness analysis in the end of Section 5.2, and added a new experiment accordingly. We hope that this addresses the concern of the reviewer.

In addition, the experimental data set seems to be a little small, so it is recommended to replace it with a larger data set.

We have replaced all experiments with experiments on a larger dataset. We have also extended the batch sizes used for runtime comparison in Fig 4
